# Epstein-Barr Virus and miRNAs: Partners in Crime in the Pathogenesis of Multiple Sclerosis?

**DOI:** 10.3389/fimmu.2019.00695

**Published:** 2019-04-03

**Authors:** Asma Hassani, Gulfaraz Khan

**Affiliations:** Department of Microbiology and Immunology, College of Medicine and Health Sciences, United Arab Emirates University, Al Ain, United Arab Emirates

**Keywords:** miRNA, post-transcriptional regulation, multiple sclerosis, EBV, immune response

## Abstract

MicroRNAs (miRNAs) are small non-coding RNAs that modulate gene expression post transcriptionally. In healthy individuals, miRNAs contribute to maintaining gene expression homeostasis. However, the level of miRNAs expressed is markedly altered in different diseases, including multiple sclerosis (MS). The impact of such changes is being investigated, and thought to shape the immune system into the inflammatory autoimmune phenotype. Much is yet to be learned about the contribution of miRNAs in the molecular pathology of MS. Epstein-Barr virus (EBV) infection is a major risk factor for the development of MS. EBV encodes more than 40 miRNAs, most of which have been studied in the context of EBV associated cancers. These viral miRNAs regulate genes involved in cell apoptosis, antigen presentation and recognition, as well as B cell transformation. If EBV infection contributes to the pathology of MS, it is plausible that EBV miRNAs may be involved. Unfortunately, there are limited studies addressing how EBV miRNAs are involved in the pathogenesis of MS. This review summarizes what has been reported regarding cellular and viral miRNA profiles in MS and proposes possible interactions between the two in the development of MS.

## Introduction

To fine tune the biological functions of a living organism, cells employ multiple cellular machineries to regulate gene expression. One of these vital machineries is the recruitment of short dsRNAs, known as microRNAs (miRNAs) to repress the target mRNAs post-transcriptionally (1). Although miRNAs play a crucial role in development, differentiation, and maintaining physiological homeostasis in many body systems, including the immune, lymphatic, and nervous systems (1–4), they also appear to contribute to the development of a wide range of diseases such as neoplasia and oncogenesis (5), autoimmunity (6, 7), and neurodegeneration (8, 9). The finding that some viruses can produce miRNAs, led to the notion that these small RNAs may also play a role in viral infection (10, 11). Moreover, miRNAs have become increasingly recognized as potential tools for monitoring response to therapy (12), disease biomarkers (13), and promising therapeutic targets (14, 15).

As the name suggests, miRNAs are small RNAs (up to 25 nucleotides in length) (16), most of which have evolved to become conserved sequences between different species (17, 18). The majority of miRNAs are polyadenylated and capped, and transcripts of RNA polymerase II (19). miRNAs form stem loop structures with a small sequence complementary to the target mRNA. The microprocessor Drosha (RNase III endonuclease), the dsRNA-binding protein DGCR8 and certain helicases (p68 and p70) act together to trim the primary miRNA into 70-nucleotide long precursor (premature) miRNA with 2-nucleotide overhang at the 3′ end only (20, 21). Exportin-5 and its cofactor Ran-GTP are responsible for shuttling nascent miRNAs from the nucleus out to the cytoplasm (22). These miRNAs encounter a complex, known as RISC (RNA-induced silencing complex) in the cytoplasm. RISC, an assembly of Dicer (RNase III) and other proteins including Argonaute, further processes miRNAs to produce the mature product with 2 overhangs, one at each end (21, 23). The two strands of miRNAs separate, one preferentially remains as a guide strand to bind its semi-complementary (i.e., ~6–8 nucleotide complementarity) mRNA target, usually at the 3′untranslated region (UTR) of mRNA, and the other (passenger) strand is degraded. The guide strand complexes with RISC to block translation of target mRNA into protein (24, 25). This inhibition of translation results from miRNA's ability to orchestrate mRNA de-adenylation and de-capping, and thus decreasing the stability of mRNA, and facilitating its degradation (26). One miRNA molecule can target many genes, and one mRNA can be targeted by multiple miRNAs, making miRNAs a part of complex cellular transcriptome hub. Nevertheless, the influence of miRNA action on protein synthesis is relatively small (1). This can be explained by the fact that many miRNAs function by inhibiting mRNA translation into protein rather than degrading mRNA itself, and it takes several binding sites on the target mRNA to be occupied by miRNAs for an efficient inhibition of translation to take place (27).

## Multiple Sclerosis and Disrupted miRNA Profiles

Multiple sclerosis (MS) is the number one cause of neurological (non-traumatic) disability in young adults worldwide (28). It is characterized with damage to myelin in the central nervous system (CNS). Myelin sheaths wrap around axons in the CNS, and play an essential role in speeding up the propagation of electrical impulses. Severely injured myelin sheaths can decelerate impulse conductance, ultimately impairing axonal tracts causing disability (29, 30). In MS, prolonged inflammation and extensive loss of myelin disturb the integrity of the white matter, leading to the formation of multiple focal lesions (also known as plaques) (31). Based on the clinical course, MS is divided into 4 subtypes: relapsing-remitting (RRMS), secondary progressive (SPMS), primary progressive (PPMS) and relapsing progressive (RPMS) (32).

The cause of MS remains unknown. However, the observation that the concordance rate of MS in monozygotic twins ranges between ~6 and 30%, depending on the geographical region (33–35), indicates that MS is a complex multifactorial disease involving both genetic and environmental etiological factors. Several gene loci have been associated with MS, but repeatedly confirmed evidence points to immune-associated HLA class II locus on chromosome 6p21.3; linked to HLA-DR2 haplotype (36). In particular, HLA-DRB1^*^1501 has the strongest link to MS risk. However, the precise mechanism linking MS to HLA-DRB1^*^1501 remains enigmatic (37). HLA class II molecules are surface glycoproteins which participate in cognate interaction between CD4^+^ T cells and antigen presenting cells (APCs). These molecules are polymorphic, and make up the antigenic epitope-binding groove. In addition to HLA class II region, HLA class I locus has also been linked to MS risk (37). Moreover, epigenetic changes have also been proposed to influence a person's risk of developing MS. The impact of epigenetics arises from the possibility that gene expression can be partially governed by chromatin and DNA modifications. Cell division can facilitate the transmission of these modifications, whether they are MS-predisposing or protecting against MS modifications, from parents to their progeny. DNA modifications do not alter the sequence of an individual's DNA. However, epigenetic modification of DNA taking place early in life has the potential to alter the available amount of certain gene products, and the possibility of events related to polymorphisms of specific genes. Epigenetics can exert their impact on many cells from different tissues or cells from one given tissue. It is not possible to demonstrate the effect of epigenetics on MS risk as a stand-alone cause. To understand epigenetic effects, one should consider the role HLA plays in MS risk. This role could be small in some individuals and substantial in others, depending on exposure to and interaction with certain environmental agents and extent of epigenetic modifications (37).

Several studies have examined the profile of cellular miRNAs in MS to identify potential disease mechanisms and markers, and design targeted therapies. To determine whether miRNA profile differs between MS patients and healthy controls, two recent studies used online databases and optimized bioinformatics tools, including integrative miRNA-mRNA interaction hubs (38, 39). They validated the *in silico* predictions using microarray and qPCR analysis of whole blood samples from MS patients and normal controls. The expression level of up to 45 miRNAs and 621 target mRNAs was found to be significantly disrupted (39). Although the sample size used in one study (39) was small and resulted in conflicting outcome between microarray and qPCR analysis of some variables, the study nevertheless showed significantly dysregulated miR-30a (upregulated), miR-20a, miR-20b, miR-211, and miR-93 (downregulated) in the circulation of MS patients. These miRNAs have been implicated in regulating transcripts involved in the immune response (39). Indeed, miR-30a together with other miRNAs such as miR-345 and miR-221-3p, have been shown to be linked to B cell activity (40) and appear to be upregulated in MS (41). The other study revealed that the expression level of miR-16, miR-24, miR-137 and miR-181 was most significantly affected in MS patients (38). Although the expression of miR-181, which is believed to be linked to activation and differentiation of B cells, was found in this study to be upregulated in MS patients, Sievers and colleagues reported that miR-181 is downregulated in non-treated MS (42). This suggests that the dysregulated profile of miRNAs in MS is affected by disease activity and/or by disease modifying drugs.

**Figure 1:**
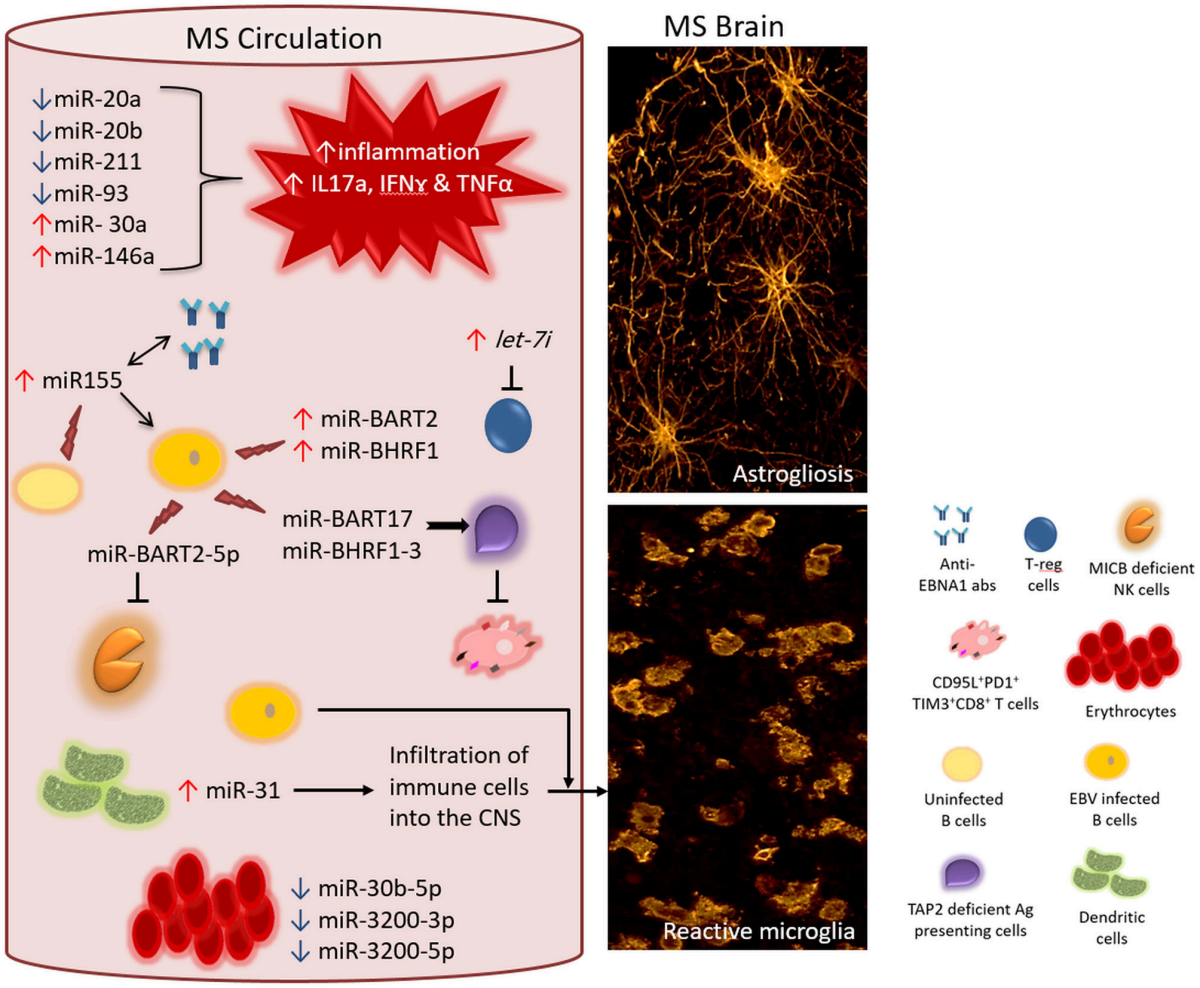
Proposed interaction between EBV encoded miRNAs and cellular miRNAs in MS. In the circulation of MS patients, the level of different cellular miRNAs is dysregulated-either significantly upregulated (↑) or downregulated (↓) including miR-20a, miR-20b, miR-211, miR-93, miR-30a, and miR-146a. Disrupted homeostasis of miRNAs impacts the levels of target mRNAs of genes involved in the local immune response. Consequently, a response that is skewed toward the inflammatory phenotype dominates (for instance increased level of IL17a, IFNγ, and TNFα mRNAs and their gene products). Moreover, the increased level of MS exosomal *let-7i* results in the suppression of regulatory T cells, which otherwise counteract the action of inflammatory subsets of T cell. Circulating B cells (non-infected) and other cellular sources of miR-155 contribute to the elevated levels of miR-155, which is associated with increased titres of anti-EBNA1 antibodies in the circulation. It is interesting to know whether these two biomarkers act directly in a positive feedback loop. Additionally, increased levels of miR-155 directly or indirectly boosts the levels of EBV encoded miR-BART2 and miR-BHRF1 clusters, both of which support the survival of EBV infected B cells. While dysregulated cellular miRNAs feed the ongoing inflammation in MS, EBV encoded miRNAs protect surviving EBV infected cells from host antiviral immune response. One mechanism of evading immune response is by impairing the capacity of antigen presenting cells. EBV encoded miR-BHRF1-3 and miR-BART17 target TAP2 mRNA resulting in downregulation of TAP2 protein. This will diminish the event of processing and presenting EBV viral antigens on class I^+^ cells to EBV-specific CD8^+^T cells. In MS, anti-EBV CD8^+^T cells are chronically activated and functionally exhausted expressing the inhibitory molecules PD1 and TIM3. Within the same context, EBV encoded miR-BART2-5p target MICB mRNA leading to the compromised functionality of anti-EBV NK cells. The few surviving EBV infected cells may potentially influence the inflammatory population in the circulation and/or cross disrupted CNS barriers. The level of cellular miR-31 rises in dendritic cells increasing the potential of infiltrating the brain and spinal cord by different immune cells including the few EBV infected cells. In MS circulation, the expression of erythrocytes miR-30b-5p, miR-3200-3p and miR-3200-5p is increased, making them a useful biomarker for monitoring disease activity. All together, these events contribute to MS pathology hallmarks such as reactive gliosis elicited by activated microglia and astrocytes (right panel).

Not only does miRNA profile differ between MS and non-MS individuals in the blood, but it also differs in the brain itself, the organ mainly affected in MS. Junker and coauthors (43) compared the levels of miRNA expression in MS brain samples from that in non-MS control brain samples. The expression of up to 22 miRNAs were found preferentially and significantly altered in MS active and inactive lesions compared to non-MS (non-demyelinative) white matter. Although few of these miRNAs were downregulated in MS brain tissues, most of them were highly upregulated with ~2–15-fold increase. To investigate the cellular source of dysregulated miRNAs in MS, studies examined different body tissues. For instance, in MS injured CNS, astrocytes were reported to express high levels of several miRNAs, including miR-155, which was shown to target the 3′ UTR of CD47. The markedly increased expression of miR-155, along with other miRNAs such as miR-34a and miR-326 would result in noticeable decline in the expression of CD47, an inhibitor of phagocytosis. Thus promoting myelin engulfment by phagocytes in MS lesions–particularly in active plaques (43). miR-155 appears to be of great importance in MS pathology (Figure 1). A study carried out on Sardinian population, reported a 7-fold increase in the expression of this miRNA in MS patients compared to sex-matched healthy controls (44). The elevated expression of miR-155 in MS patients correlated with an increase in circulating humoral response to Epstein-Barr virus nuclear antigen 1 (EBNA1). Interestingly, after 6-months of treatment with natalizumab, a monoclonal antibody against the cell adhesion molecule α4β7 integrin, the level of miR-155 and EBNA1 IgG titer in the blood of MS patients significantly dropped compared to no difference observed in the blood of healthy controls. However, an earlier study reported increased levels of EBV miRNAs, and not miRNAs of JC and BK viruses, as an effect of natalizumab treatment (42). Besides lowering the level of miR-155, this negative regulatory effect of natalizumab therapy was also seen on IL-17a, IFN-γ, and TNFα mRNAs (44). The question whether the dysregulated level of miR-155 is directly associated with anti-viral humoral response in MS has not been addressed. Consistent with these observations, studies using miR-155 knockout mice, revealed that these animals are resistant or develop a clinically and histologically mild form of experimental autoimmune encephalomyelitis (EAE) when challenged with encephalitogenic self-peptides derived from oligodendrocyte glycoprotein (45, 46). It appears that the roles played by miR-155 support T cell-driven inflammation and autoimmunity. Another example of cellular source of dysregulated miRNAs is dendritic cells. In the animal model of MS, dendritic cells were found to express a high level of miR-31, which downstream aided the CNS infiltrating myelin-attacking immune cells (47).

Another source of MS disrupted miRNAs is believed to be erythrocytes. A study investigating the miRNA profile in erythrocytes purified from whole blood from RRMS patients and healthy controls revealed that only a handful of erythrocyte miRNAs, namely miR-30b-5p, miR-3200-3p and miR-3200-5p, could be linked to MS (48) (Figure 1).

In addition to cellular miRNAs, exosomal miRNAs are also believed to contribute to the pathogenesis of MS. For instance, Kimura and coauthors suggested that the pattern of exosomal miRNAs circulating in the blood of MS patients is unique in that, the miRNA *let-7i* is markedly increased in MS patients and it could negatively affect the differentiation of CD4^+^T cells into the regulatory Foxp3^+^CD4^+^ Treg cells. The study showed that the inhibitory effect of *let-7i* was mediated through blocking the expression of transforming growth factor β receptor 1 (TGFBR1) and insulin-like growth factor 1 receptor (IGF1R) (49). This decreased polarization of T cells toward the regulatory phenotype could throw the immune response out of balance, leaving the action of inflammatory immune cells with little checkpoints (Figure 1). Moreover, it is proposed that MS patients have distinct genetic variants of miRNAs. An example of this is the genotype (GC+CC) of the variant rs2910164 in miR-146a, which has been shown to be associated with higher risk of MS disease progression. This genotype was also shown to act in synergism with EBV infection related parameters. Consequently, individuals with this genotype of miR-146a and an elevated baseline humoral response to EBNA1 and EBNA2, or history of infectious mononucleosis (IM) are at a greater risk than the general population of developing MS or experiencing an exacerbation of disease course (50). Supporting this finding, the knockout of miR-146a in murine MS model was found to ameliorate inflammation, as opposed to wild type murine MS model (51).

As pointed out above, there is an indication that dysregulated cellular miRNAs may interact with the immune system and viral infection, particularly EBV infection, in contributing to the pathogenesis of MS. Unfortunately, the details of how these potential risk factors interact, remain unknown. This highlights the need to explore the behavior of cellular miRNAs in different body compartments at different time points in the development of MS (early MS- progressive MS), and investigate possible links between regulatory miRNAs and EBV infection. However, this endeavor is probably more challenging and complex given the fact that one miRNA molecule can have various effects on multiple targets in different settings and different tissues. This is even more complicated due to the varying expression profiles of miRNAs.

## EBV, EBV miRNAs, and MS

Over 40 environmental risk factors have been studied in association with MS, but EBV infection has the strongest sero-epidemiological evidence (52). EBV is a member of the *Herpesviridae* family of large DNA viruses (53, 54). EBV is highly prevalent in the human population, infecting more than 90% of people worldwide. Although EBV infection in the vast majority of people is largely harmless, it has been associated with several malignancies and the incidence of global deaths due to EBV-related malignancies has been rising (55).

*In vitro* EBV infection of B lymphocytes, leads to immortalization of these cells into lymphoblastoid cell lines (LCLs). In LCLs, EBV genome circularizes into an episome, and the virus establishes a latent infection without killing the cell (54). Although EBV genome is large and can code for over 80 genes, less than a dozen are actually expressed at the protein level in LCLs. These include, 6 EBV nuclear antigens (EBNA1,-2,-3A,-3B,-3C, -LP) and 3 latent membrane proteins (LMP-1,-2A and -2B). Each one of the 6 EBNAs is encoded by one distinct mRNA. Either Cp or Wp promoters (positioned in *Bam*HI C and W region) express a “rightward” key transcript that is more than 100 kb long (56, 57). The differential splicing of this transcript produces EBNA mRNAs. During early stages of EBV infection of B cells, Cp promoter is transactivated by EBNA1 and EBNA2. Different promoters located in the *Bam*HI N region express LMP transcripts. LMP promoters are transactivated by EBNA2 (58). The function of latent genes products appear to affect differentiation and proliferation of host cells, providing EBV with the potential of cellular transformation and coping with different events in the life cycle of B cells (59). Although most of EBV latent genes are translated into protein products, some are not meant to be translated, including EBV-encoded RNA, EBER-1 and -2, and BARTs (BamHI A rightward transcripts) (60).

EBV encodes for more than 40 miRNAs originating from 25 precursor transcripts. BART and BHRF1 (Bam HI fragment H rightward open reading frame 1) regions encode clusters of EBV miRNAs. The profile of EBV miRNAs has been investigated mainly in EBV immortalized cell lines (61) and EBV-associated tumor biopsies (62). The levels of EBV miRNAs expressed in tumor biopsies were observed to be higher (over 100-fold) than that in EBV cell lines. This suggests that EBV miRNAs could have more gene targets –to be regulated- *in vivo* than in *in vitro* cultures (63). Additionally, the expression of miR-BHRF1 clusters is less frequent than that of miR-BART clusters, which are detected in cells with different EBV latency programs (62).

Various roles have been implicated for EBV miRNAs, including, inhibition of apoptosis, promoting B cell survival, inducing B cell transformation, and most importantly, in evading immune recognition and antiviral attack (64). However, there remains a big gap in identifying whether and how EBV miRNAs act in promoting the pathogenesis of diseases other than carcinomas and EBV IM. Indeed, if EBV is involved the pathogenesis of autoimmune diseases such as MS, looking at how EBV miRNAs engage in the disease process could offer new insights into how MS develops and potential strategies for treatment and prevention. Probably one of the most indispensable functions of EBV-encoded miRNAs is immune evasion. Albanese et al (65) compared two types of cultures of *in vitro* EBV infection of human B cells. In one culture, primary B cells were infected with wild-type EBV expressing viral miRNAs, while in the other culture, B cells were infected with virus deficient of miRNAs. Upon EBV infection, B cells in both cultures expanded. When auto-CD8^**+**^ T cells were introduced into the cultures, cells infected with miRNAs-deficient virus were more susceptible to cell death compared to cells infected with wild-type virus. Further investigations revealed that EBV miRNA expressing culture significantly reduced the cytotoxicity (indicated by IFN-γ secretion) and clonal expansion of anti-EBV CD8^**+**^ T cells, and thus protecting infected B cells and sustaining their viability (65). Using *in silico* prediction tools followed by *in vitro* transfection system and luciferase reporter assays, revealed that EBV miRNAs (miR-BHRF1-3 and miR-BART17) targeted the 3′ end of the *TAP2*, causing inhibition of expression of TAP2 protein. TAP2 is involved in antigen processing and presentation on MHC class I^+^ cells. Thus, this could explain how EBV may evade immune recognition (Figure 1). Within the same context, the expression of HLA-B^*^07, HLA-B^*^08, and HLA-B^*^40 haplotypes was also shown to be lower in EBV miRNA expressing culture compared to EBV miRNA-deficient culture. Interestingly, the level of HLA-A^*^02, associated with reduced risk of MS (66–68), in EBV miRNA expressing culture was shown to be comparable to that in EBV miRNA deficient culture (65). The same group demonstrated in an earlier study that EBV also makes use of its miRNAs to escape recognition by CD4^+^ T cells and attenuate viral gene processing and presentation on class II molecules expressing cells (69). EBV miRNAs are also thought to modulate the expression of inflammatory cytokines. Using luciferase reporter assays and Western blotting, it has been shown that EBV encoded miR-BHRF1-2-5p targets the 3′UTR of IL-1 receptor 1 (IL-1R1). This was reflected in the reduced production of the protein (i.e., decreased expression of IL-1R), which is physiologically involved in alerting the immune system for presence of viral infection (70).

Collectively, these studies suggest an intimate relationship between EBV miRNAs and the immune system, with the latter being on a direct intersection with MS. To investigate the effect of EBV infection on the immune system in MS, a recent study characterized, phenotypically and functionally, T cells derived from MS white matter lesions in 27 MS cases from the Dutch population (71). The investigators carried out a comprehensive analysis involving different body compartments (blood, CSF, and white matter/brain parenchyma). Flow cytometry analysis showed that brain lesion derived T cells were frequently cytotoxic (granzyme B secreting) CD8^**+**^ T cells of effector memory phenotype. These cells were also found to be chronically activated, expressing CD95L and the inhibitory molecules PD1 and TIM3, and had increased reactivity against EBV auto-LCLs, but not against 7 potential self-antigens related to MS (71). In spite of their reactivity against EBV, CD8^**+**^ T cells appear to have an impaired antiviral response, particularly in the periphery in MS (72–74). The increased expression of the inhibitory surface molecule PD1 by on CD8^**+**^ T cells results in a markedly reduced cytolytic activity against EBV infected cells (72), in particular latently infected cells (74).

How EBV infection influences the immune system in MS was also studied in a marmoset model of EAE. EBV infected B cells are thought to serve as professional antigen presenting cells that uptake myelin derived antigens, process and present them to autoreactive CD8^**+**^ T cells, triggering perturbed local immune response (75). Uninfected B cells on the other hand, capture myelin antigens, which undergo processing by cathepsins in the lysosome, but never end up displaying them on cell surface because self-antigens are degraded intracellularly (76). In line with this, EBV encoded miRNA clusters, BHRF1-2, BART1-5p, BART1-3p, and BART2-5p, were shown to target multiple genes involved in antigen processing, including *CTSB* (coding for cathepsin B), *LGMN* (coding for asparagine endopeptidase) and *IFI30* (coding for γ interferon-inducible lysosomal thiol reductase) (70). It would be interesting to know whether EBV miRNAs has the ability to discriminate between mRNAs of genes involved in processing viral antigens and those of genes involved in processing self-antigens. Indeed, in marmoset model, RNA sequencing revealed that EBV infection alters the expression of genes coding for molecules required for (1) appropriate antigen processing and presentation, and (2) providing immune cells with surface costimulatory signals (73). Some of the markers that have been reported to be downregulated upon EBV infection include CCR7; a T cell surface molecule needed for homing to secondary lymphoid tissues; CD27, a member of TNFR family, and CCR6 and IL-23R, a chemokine and cytokine receptor, respectively (73). These observations support the notion that EBV infection greatly influences the immune response in MS and a more detailed picture is needed to fully uncover the link between the virus and MS.

Another link between EBV infection and MS is the history of IM. IM results from delayed primary EBV infection, and several reports indicate that it is a major risk factor for the development of MS (77–82). By contrast, very few studies have examined EBV miRNAs in IM. A study that investigated the profile of EBV miRNAs in children post kidney transplantation and compared it to children with IM, found no significant correlation between EBV copy number and the number of EBV encoded miRNAs expressed in plasma (83). Nevertheless, transplant recipients who had no detectable levels of EBV load had lower levels of EBV miRNAs, compared to transplant recipients with chronically elevated EBV load. The plasma level of EBV miRNAs in non-transplant IM patients was as high as that in transplant recipients with chronically elevated peripheral EBV load. Of note, only IM patients had detectable levels of the lytic EBV miR-BHRF1-2-3p and miR-BHRF1-1. This study was limited by the lack of healthy controls for comparison and the small sample size (83). Additionally, most (87.5%) of IM patients and (75%) transplant recipients with chronically elevated viral load had detectable level of EBV miR-BART2-5p in plasma, compared to only 58.3% of transplant recipients with no detectable viral load (83). The regulatory role of EBV miR-BART2 was described in targeting the 3′UTR of EBV *BALF5* coding for BALF5, an EBV DNA polymerase. The resulting inhibition of BALF5 leads to decreased viral shedding (84). EBV miR-BART2-5p cluster was also reported to target 3′UTR of *MICB* gene, leading to suppression of NK cells function (85) (Figure 1). Hence, EBV miRNAs again seem to help EBV infected cells evade the immune system.

Another study in pediatric population focused on quantifying cellular miRNAs and EBV encoded miRNAs in IM patients and EBV seropositive healthy controls (86). Of the 44 EBV miRNAs tested in this study, 41 were detected at elevated levels in early phase of IM, compared to only 11 in control group. Generally, the plasma levels and B cell expression of EBV miRNAs decreased with time in the IM group. Of the 84 cellular miRNAs tested, 40 were expressed at least 2-fold more in early IM than in healthy controls. The expression of most cellular miRNAs in CD8^**+**^ T cells was found to drop with time. In contrast, the B cell expression of most cellular miRNAs, associated with immune response and cell differentiation, was found to increase with time (86). Among these cellular miRNAs are miR-155, miR-137, miR-181, and miR-146a, all of which were reported to be dysregulated in MS (38, 39, 44, 45, 87). Moreover, a positive trend was observed between B cell miR-155 and miR-181 and EBV miR-BART2 and miR-BHRF1 clusters (86). EBV miR-BHRF1 has been described in EBV triggered cell transformation (88) and promoting EBV infected B cell survival *in vitro* (89). However, little is known about how these altered viral miRNAs in IM modulate the expression of cellular miRNAs, and how their interaction can make IM a risk factor for the development of MS. Thereby, it is safe to propose that EBV encoded miRNAs and dysregulated cellular miRNAs may interact, regardless of which depends on which, and contribute to the pathology seen in MS (Figure 1). Indeed, it appears that the dysregulated levels of cellular miRNAs in MS can promote EBV infection in MS and serve the surviving infected cells (90).

## Conclusion

Of the environmental factors, substantial amount of data indicates that EBV is directly or indirectly involved in the pathogenesis of MS (91–95). Moreover, cellular, exosomal, plasma and erythrocyte miRNAs profiles indicate that these profiles are disrupted in MS patients, compared to healthy controls (15, 48, 49, 96, 97). Although a large number of studies have investigated the significance of EBV encoded miRNAs in altering the levels of viral and cellular genes in EBV associated malignancies (88, 98), much less is known in the context of MS. On the one hand, the disrupted homeostasis of cellular miRNAs in MS appears to serve the ongoing inflammation linked to the disease, whilst on the other hand, they may aid in supporting the survival of EBV infected cells by positively influencing viral miRNAs that function in evading anti-EBV immune response. Dysregulated cellular miRNAs may also pave the way for CNS infiltration by inflammatory cells and EBV infected cells. It is reasonable to think that EBV miRNAs in MS CNS can also impact CNS resident cells, particularly those involved with MS pathology. Thus, investigating the targets and role of EBV miRNAs in MS, mainly in peripheral blood, CSF, brain, and CNS draining lymph nodes, could shed light on disease mechanism not yet explored.

## Author Contributions

All authors listed have made a substantial, direct and intellectual contribution to the work, and approved it for publication.

### Conflict of Interest Statement

The authors declare that the research was conducted in the absence of any commercial or financial relationships that could be construed as a potential conflict of interest.

## References

[1] Bartel DP. MicroRNAs: target recognition and regulatory functions. Cell. (2009) 136:215–33. 10.1016/j.cell.2009.01.002 19167326 PMC3794896

[2] Breving K Esquela-Kerscher A. The complexities of microRNA regulation: mirandering around the rules. Int J Biochem Cell Biol. (2010) 42:1316–29. 10.1016/j.biocel.2009.09.016 19800023

[3] Forero A So L Savan R. Re-evaluating strategies to define the immunoregulatory roles of miRNAs. Trends Immunol. (2017) 38:558–66. 10.1016/j.it.2017.06.001 28666937 PMC5551433

[4] Lee HM Nguyen DT Lu LF. Progress and challenge of microRNA research in immunity. Front Genet. (2014) 5:178. 10.3389/fgene.2014.00178 24971086 PMC4053854

[5] Mazan-Mamczarz K Gartenhaus RB. Role of microRNA deregulation in the pathogenesis of diffuse large B-cell lymphoma (DLBCL) Leuk Res. (2013) 37:1420–8. 10.1016/j.leukres.2013.08.020 24054860 PMC3856880

[6] Amici SA Dong J Guerau-de-Arellano M. Molecular mechanisms modulating the phenotype of macrophages and microglia. Front Immunol. (2017) 8:1520. 10.3389/fimmu.2017.01520 29176977 PMC5686097

[7] Vinuesa CG Rigby RJ Yu D. Logic and extent of miRNA-mediated control of autoimmune gene expression. Int Rev Immunol. (2009) 28:112–38. 10.1080/08830180902934909 19811318

[8] Fransquet PD Ryan J. Micro RNA as a potential blood-based epigenetic biomarker for Alzheimer's disease. Clin Biochem. (2018) 58:5–14. 10.1016/j.clinbiochem.2018.05.020 29885309

[9] Quinlan S Kenny A Medina M Engel T Jimenez-Mateos EM. MicroRNAs in neurodegenerative diseases. Int Rev Cell Mol Biol. (2017) 334:309–43. 10.1016/bs.ircmb.2017.04.002 28838542

[10] Burke JM Bass CR Kincaid RP Sullivan CS. Identification of tri-phosphatase activity in the biogenesis of retroviral microRNAs and RNAP III-generated shRNAs. Nucleic Acids Res. (2014) 42:13949–62. 10.1093/nar/gku1247 25428356 PMC4267658

[11] Kincaid RP Sullivan CS. Lessons learned from *in vivo* studies of a viral noncoding RNA. mSphere. (2016) 1:e00026–16. 10.1128/mSphere.00026-16 27301787 PMC4863581

[12] Hon KW Abu N Ab Mutalib NS Jamal R. miRNAs and lncRNAs as predictive biomarkers of response to FOLFOX therapy in colorectal cancer. Front Pharmacol. (2018) 9:846. 10.3389/fphar.2018.00846 30127741 PMC6088237

[13] Akerman L Casas R Ludvigsson J Tavira B Skoglund C. Serum miRNA levels are related to glucose homeostasis and islet autoantibodies in children with high risk for type 1 diabetes. PLoS ONE. (2018) 13:e0191067. 10.1371/journal.pone.0191067 29346396 PMC5773164

[14] Ou J Kou L Liang L Tang C MiR-375 attenuates injury of cerebral ischemia/reperfusion via targeting Ctgf. Biosci Rep. (2017) 37:BSR20171242 10.1042/BSR20171242 29187583 PMC5741829

[15] Saenz-Cuesta M Alberro A Munoz-Culla M Osorio-Querejeta I Fernandez-Mercado M Lopetegui I. The first dose of fingolimod affects circulating extracellular vesicles in multiple sclerosis patients. Int J Mol Sci. (2018) 19:E2448. 10.3390/ijms19082448 30126230 PMC6121302

[16] Berezikov E. Evolution of microRNA diversity and regulation in animals. Nat Rev Genet. (2011) 12:846–60. 10.1038/nrg3079 22094948

[17] Lee CT Risom T Strauss WM. Evolutionary conservation of microRNA regulatory circuits: an examination of microRNA gene complexity and conserved microRNA-target interactions through metazoan phylogeny. DNA Cell Biol. (2007) 26:209–18. 10.1089/dna.2006.0545 17465887

[18] Zhang R Su B. Small but influential: the role of microRNAs on gene regulatory network and 3′UTR evolution. J Genet Genomics. (2009) 36:1–6. 10.1016/S1673-8527(09)60001-1 19161940

[19] Lee Y Kim M Han J Yeom KH Lee S Baek SH. MicroRNA genes are transcribed by RNA polymerase II. Embo J. (2004) 23:4051–60. 10.1038/sj.emboj.7600385 15372072 PMC524334

[20] Ha M Kim VN. Regulation of microRNA biogenesis. Nat Rev Mol Cell Biol. (2014) 15:509–24. 10.1038/nrm3838 25027649

[21] Winter J Jung S Keller S Gregory RI Diederichs S. Many roads to maturity: microRNA biogenesis pathways and their regulation. Nat Cell Biol. (2009) 11:228–34. 10.1038/ncb0309-228 19255566

[22] Yi R Qin Y Macara IG Cullen BR. Exportin-5 mediates the nuclear export of pre-microRNAs and short hairpin RNAs. Genes Dev. (2003) 17:3011–6. 10.1101/gad.1158803 14681208 PMC305252

[23] Blahna MT Hata A. Regulation of miRNA biogenesis as an integrated component of growth factor signaling. Curr Opin Cell Biol. (2013) 25:233–40. 10.1016/j.ceb.2012.12.005 23312066 PMC4429755

[24] Romero-Cordoba SL Salido-Guadarrama I Rodriguez-Dorantes M Hidalgo-Miranda A. miRNA biogenesis: biological impact in the development of cancer. Cancer Biol Ther. (2014) 15:1444–55. 10.4161/15384047.2014.955442 25482951 PMC4622859

[25] Schwarz DS Hutvagner G Du T Xu Z Aronin N Zamore PD. Asymmetry in the assembly of the RNAi enzyme complex. Cell. (2003) 115:199–208. 10.1016/S0092-8674(03)00759-1 14567917

[26] Wu L Fan J Belasco JG. MicroRNAs direct rapid deadenylation of mRNA. Proc Natl Acad Sci USA. (2006) 103:4034–9. 10.1073/pnas.0510928103 16495412 PMC1449641

[27] Pillai RS Bhattacharyya SN Filipowicz W. Repression of protein synthesis by miRNAs: how many mechanisms? Trends Cell Biol. (2007) 17:118–26. 10.1016/j.tcb.2006.12.007 17197185

[28] Frischer JM Weigand SD Guo Y Kale N Parisi JE Pirko I. Clinical and pathological insights into the dynamic nature of the white matter multiple sclerosis plaque. Ann Neurol. (2015) 78:710–21. 10.1002/ana.24497 26239536 PMC4623970

[29] Love S. Demyelinating diseases. J Clin Pathol. (2006) 59:1151–9. 10.1136/jcp.2005.031195 17071802 PMC1860500

[30] Quarles RH Morell P Basic neurochemistry: molecular, cellular and medical aspects. In: American Society for Neurochemistry. Siegel BW Agranoff RW Albers SK Fisher MD Uhler, editors. Philadelphia: Lippincott-Raven (1999).

[31] Popescu BF Pirko I Lucchinetti CF. Pathology of multiple sclerosis: where do we stand? Continuum. (2013) 19:901–21. 10.1212/01.CON.0000433291.23091.65 23917093 PMC3915566

[32] Compston A Coles A. Multiple sclerosis. Lancet. (2008) 372:1502–17. 10.1016/S0140-6736(08)61620-7 18970977

[33] Multiple sclerosis in 54 twinships: concordance rate is independent of zygosity French research group on multiple sclerosis. Ann Neurol. (1992) 32:724–7. 10.1002/ana.410320604 1471862

[34] Ristori G Cannoni S Stazi MA Vanacore N Cotichini R Alfo M. Multiple sclerosis in twins from continental Italy and Sardinia: a nationwide study. Ann Neurol. (2006) 59:27–34. 10.1002/ana.20683 16240370

[35] Wade BJ. Spatial analysis of global prevalence of multiple sclerosis suggests need for an updated prevalence scale. Mult Scler Int. (2014) 2014:124578. 10.1155/2014/124578 24693432 PMC3945785

[36] Baranzini SE. Revealing the genetic basis of multiple sclerosis: are we there yet? Curr Opin Genet Dev. (2011) 21:317–24. 10.1016/j.gde.2010.12.006 21247752 PMC3105160

[37] Hoppenbrouwers IA Hintzen RQ. Genetics of multiple sclerosis. Biochim Biophys Acta. (2011) 1812:194–201. 10.1016/j.bbadis.2010.09.017 20933079

[38] Ehya F Abdul Tehrani H Garshasbi M Nabavi SM. Identification of miR-24 and miR-137 as novel candidate multiple sclerosis miRNA biomarkers using multi-staged data analysis protocol. Mol Biol Res Commun. (2017) 6:127–40. 10.22099/mbrc.2017.24861.1256 29071282 PMC5640895

[39] Yang Q Pan W Qian L. Identification of the miRNA-mRNA regulatory network in multiple sclerosis. Neurol Res. (2017) 39:142–51. 10.1080/01616412.2016.1250857 27809691

[40] Huang Q Xiao B Ma X Qu M Li Y Nagarkatti P. MicroRNAs associated with the pathogenesis of multiple sclerosis. J Neuroimmunol. (2016) 295–296:148–61. 10.1016/j.jneuroim.2016.04.014 27235360

[41] Freiesleben S Hecker M Zettl UK Fuellen G Taher L. Analysis of microRNA and gene expression profiles in multiple sclerosis: integrating interaction data to uncover regulatory mechanisms. Sci Rep. (2016) 6:34512. 10.1038/srep34512 27694855 PMC5046091

[42] Sievers C Meira M Hoffmann F Fontoura P Kappos L Lindberg RL. Altered microRNA expression in B lymphocytes in multiple sclerosis: towards a better understanding of treatment effects. Clin Immunol. (2012) 144:70–9. 10.1016/j.clim.2012.04.002 22659298

[43] Junker A Krumbholz M Eisele S Mohan H Augstein F Bittner R. MicroRNA profiling of multiple sclerosis lesions identifies modulators of the regulatory protein CD47. Brain. (2009) 132:3342–52. 10.1093/brain/awp300 19952055

[44] Mameli G Arru G Caggiu E Niegowska M Leoni S Madeddu G. Natalizumab therapy modulates miR-155, miR-26a and proinflammatory cytokine expression in MS patients. PLoS ONE. (2016) 11:e0157153. 10.1371/journal.pone.0157153 27310932 PMC4911163

[45] Mycko MP Cichalewska M Cwiklinska H Selmaj KW. miR-155-3p drives the development of autoimmune demyelination by regulation of heat shock protein 40. J Neurosci. (2015) 35:16504–15. 10.1523/JNEUROSCI.2830-15.2015 26674874 PMC6605508

[46] O'Connell RM Kahn D Gibson WS Round JL Scholz RL Chaudhuri AA. MicroRNA-155 promotes autoimmune inflammation by enhancing inflammatory T cell development. Immunity. (2010) 33:607–19. 10.1016/j.immuni.2010.09.009 20888269 PMC2966521

[47] Hoye ML Archambault AS Gordon TM Oetjen LK Cain MD Klein RS. MicroRNA signature of central nervous system-infiltrating dendritic cells in an animal model of multiple sclerosis. Immunology. (2018) 155:112–22. 10.1111/imm.12934 29749614 PMC6099169

[48] Groen K Maltby VE Lea RA Sanders KA Fink JL Scott RJ. Erythrocyte microRNA sequencing reveals differential expression in relapsing-remitting multiple sclerosis. BMC Med Genomics. (2018) 11:48. 10.1186/s12920-018-0365-7 29783973 PMC5963124

[49] Kimura K Hohjoh H Fukuoka M Sato W Oki S Tomi C. Circulating exosomes suppress the induction of regulatory T cells via let-7i in multiple sclerosis. Nat Commun. (2018) 9:17. 10.1038/s41467-017-02406-2 29295981 PMC5750223

[50] Zhou Y Chen M Simpson SJr Lucas RM Charlesworth JC Blackburn N. Common genetic variation within miR-146a predicts disease onset and relapse in multiple sclerosis. Neurol Sci.(2017) 39:297–304. 10.1007/s10072-017-3177-1 29127522

[51] Martin NA Molnar V Szilagyi GT Elkjaer ML Nawrocki A Okarmus J. Experimental demyelination and axonal loss are reduced in MicroRNA-146a deficient mice. Front Immunol. (2018) 9:490. 10.3389/fimmu.2018.00490 29593734 PMC5857529

[52] Belbasis L Bellou V Evangelou E Ioannidis JP Tzoulaki I. Environmental risk factors and multiple sclerosis: an umbrella review of systematic reviews and meta-analyses. Lancet Neurol. (2015) 14:263–73. 10.1016/S1474-4422(14)70267-4 25662901

[53] Cornaby C Tanner A Stutz EW Poole BD Berges BK. Piracy on the molecular level: human herpesviruses manipulate cellular chemotaxis. J Gen Virol. (2016) 97:543–60. 10.1099/jgv.0.000370 26669819

[54] Whitley RJ. Herpesviruses. In: Medical Microbiology. University of Texas Medical Branch at Galveston. Galveston, TX: The University of Texas Medical Branch at Galveston (1996).

[55] Khan G Hashim MJ. Global burden of deaths from Epstein-Barr virus attributable malignancies 1990-2010. Infect Agent Cancer. (2014) 9:38. 10.1186/1750-9378-9-38 25473414 PMC4253616

[56] El-Sharkawy A Al Zaidan L Malki A. Epstein-barr virus-associated malignancies: roles of viral oncoproteins in carcinogenesis. Front Oncol. (2018) 8:265. 10.3389/fonc.2018.00265 30116721 PMC6082928

[57] Tao Q Young LS Woodman CB Murray PG. Epstein-Barr virus (EBV) and its associated human cancers–genetics, epigenetics, pathobiology and novel therapeutics. Front Biosci. (2006) 11:2672–713. 10.2741/2000 16720343

[58] Kieff E Rickinson AB Epstein-Barr virus and its replication. In: Fields' Virology. Griffin DE, editor. Philadelphia: Lippincott Williams and Wilkins (2001). p. 2511–2551.

[59] Masucci MG. Epstein-Barr virus oncogenesis and the ubiquitin-proteasome system. Oncogene. (2004) 23:2107–15. 10.1038/sj.onc.1207372 15021898

[60] Young LS Yap LF Murray PG. Epstein-Barr virus: more than 50 years old and still providing surprises. Nat Rev Cancer. (2016) 16:789–802. 10.1038/nrc.2016.92 27687982

[61] Pratt ZL Kuzembayeva M Sengupta S Sugden B. The microRNAs of Epstein-Barr Virus are expressed at dramatically differing levels among cell lines. Virology. (2009) 386:387–97. 10.1016/j.virol.2009.01.006 19217135 PMC2763627

[62] Yang YC Liem A Lambert PF Sugden B. Dissecting the regulation of EBV's BART miRNAs in carcinomas. Virology. (2017) 505:148–54. 10.1016/j.virol.2017.02.013 28259048 PMC5504693

[63] Chen SJ Chen GH Chen YH Liu CY Chang KP Chang YS. Characterization of Epstein-Barr virus miRNAome in nasopharyngeal carcinoma by deep sequencing. PLoS ONE. (2010) 5:e12745. 10.1371/journal.pone.0012745 20862214 PMC2942828

[64] Kuzembayeva M Hayes M Sugden B. Multiple functions are mediated by the miRNAs of Epstein-Barr virus. Curr Opin Virol. (2014) 7:61–5. 10.1016/j.coviro.2014.04.003 24814666 PMC4149930

[65] Albanese M Tagawa T Bouvet M Maliqi L Lutter D Hoser J. Epstein-Barr virus microRNAs reduce immune surveillance by virus-specific CD8+ T cells. Proc Natl Acad Sci USA. (2016) 113:E6467–75. 10.1073/pnas.1605884113 27698133 PMC5081573

[66] Hedstrom AK Lima Bomfim I Barcellos L Gianfrancesco M Schaefer C Kockum I. Interaction between adolescent obesity and HLA risk genes in the etiology of multiple sclerosis. Neurology. (2014) 82:865–72. 10.1212/WNL.0000000000000203 24500647 PMC3959752

[67] Jilek S Schluep M Harari A Canales M Lysandropoulos A Zekeridou A. HLA-B7-restricted EBV-specific CD8+ T cells are dysregulated in multiple sclerosis. J Immunol. (2012) 188:4671–80. 10.4049/jimmunol.1103100 22461701

[68] Silva AM Bettencourt A Pereira C Santos E Carvalho C Mendonca D. Protective role of the HLA-A^*^02 allele in Portuguese patients with multiple sclerosis. Mult Scler. (2009) 15:771–4. 10.1177/1352458509104588 19482867

[69] Tagawa T Albanese M Bouvet M Moosmann A Mautner J Heissmeyer V. Epstein-Barr viral miRNAs inhibit antiviral CD4+ T cell responses targeting IL-12 and peptide processing. J Exp Med. (2016) 213:2065–80. 10.1084/jem.20160248 27621419 PMC5030804

[70] Skinner CM Ivanov NS Barr SA Chen Y Skalsky RL. An epstein-barr virus MicroRNA blocks interleukin-1. (IL-1) signaling by targeting IL-1 receptor 1. J Virol. (2017) 91:e00530–17. 10.1128/JVI.00530-17 28794034 PMC5640834

[71] van Nierop GP van Luijn MM Michels SS Melief MJ Janssen M Langerak AW. Phenotypic and functional characterization of T cells in white matter lesions of multiple sclerosis patients. Acta Neuropathol. (2017) 134:383–401. 10.1007/s00401-017-1744-4 28624961 PMC5563341

[72] Cencioni MT Magliozzi R Nicholas R Ali R Malik O Reynolds R. Programmed death 1 is highly expressed on CD8(+) CD57(+) T cells in patients with stable multiple sclerosis and inhibits their cytotoxic response to Epstein-Barr virus. Immunology. (2017) 152:660–76. 10.1111/imm.12808 28767147 PMC5680058

[73] Dunham J van Driel N Eggen BJ Paul C t Hart BA Laman JD. Analysis of the cross-talk of Epstein-Barr virus-infected B cells with T cells in the marmoset. Clin Transl Immunology. (2017) 6:e127. 10.1038/cti.2017.1 28243437 PMC5311918

[74] Pender MP Csurhes PA Burrows JM Burrows SR Defective T-cell control of Epstein-Barr virus infection in multiple sclerosis. Clin Transl Immunology. (2017) 6:e126 10.1038/cti.2016.87 28197337 PMC5292561

[75] Morandi E Jagessar SA t Hart BA Gran B. EBV infection empowers human B cells for autoimmunity: role of autophagy and relevance to multiple sclerosis. J Immunol. (2017) 199:435–48. 10.4049/jimmunol.1700178 28592428

[76] t Hart BA Kap Y S An essential role of virus-infected B cells in the marmoset experimental autoimmune encephalomyelitis model. Mult Scler J Exp Transl Clin. (2017) 3:2055217317690184 10.1177/2055217317690184 28607749 PMC5466146

[77] Haahr S Koch-Henriksen N Moller-Larsen A Eriksen LS Andersen HM. Increased risk of multiple sclerosis after late Epstein-Barr virus infection: a historical prospective study. Mult Scler. (1995) 1:73–7. 10.1177/135245859500100203 9345455

[78] Hedstrom AK Lima Bomfim I Hillert J Olsson T Alfredsson L. Obesity interacts with infectious mononucleosis in risk of multiple sclerosis. Eur J Neurol. (2015) 22:578–e38. 10.1111/ene.12620 25530445 PMC4365756

[79] Lindberg C Andersen O Vahlne A Dalton M Runmarker B. Epidemiological investigation of the association between infectious mononucleosis and multiple sclerosis. Neuroepidemiology. (1991) 10:62–5. 10.1159/000110248 2062419

[80] Mameli G Madeddu G Cossu D Galleri G Manetti R Babudieri S. Immune response induced by Epstein-Barr virus and Mycobacterium avium subsp. paratuberculosis peptides in current and past infectious mononucleosis: a risk for multiple sclerosis? Eur J Neurol. (2016) 23:140–7. 10.1111/ene.12821 26453465

[81] Mameli G Madeddu G Mei A Uleri E Poddighe L Delogu LG. Activation of MSRV-type endogenous retroviruses during infectious mononucleosis and Epstein-Barr virus latency: the missing link with multiple sclerosis? PLoS ONE. (2013) 8:e78474. 10.1371/journal.pone.0078474 24236019 PMC3827255

[82] Thacker EL Mirzaei F Ascherio A. Infectious mononucleosis and risk for multiple sclerosis: a meta-analysis. Ann Neurol. (2006) 59:499–503. 10.1002/ana.20820 16502434

[83] Hassan J Dean J De Gascun CF Riordan M Sweeney C Connell J. Plasma EBV microRNAs in paediatric renal transplant recipients. J Nephrol. (2017) 31:445–51. 10.1007/s40620-017-0462-2 29185211

[84] Barth S Pfuhl T Mamiani A Ehses C Roemer K Kremmer E. Epstein-Barr virus-encoded microRNA miR-BART2 down-regulates the viral DNA polymerase BALF5. Nucleic Acids Res. (2008) 36:666–75. 10.1093/nar/gkm1080 18073197 PMC2241876

[85] Nachmani D Stern-Ginossar N Sarid R Mandelboim O. Diverse herpesvirus microRNAs target the stress-induced immune ligand MICB to escape recognition by natural killer cells. Cell Host Microbe. (2009) 5:376–85. 10.1016/j.chom.2009.03.003 19380116

[86] Gao L Ai J Xie Z Zhou C Liu C Zhang H. Dynamic expression of viral and cellular microRNAs in infectious mononucleosis caused by primary Epstein-Barr virus infection in children. Virol J. (2015) 12:208. 10.1186/s12985-015-0441-y 26634702 PMC4669648

[87] Waschbisch A Atiya M Linker RA Potapov S Schwab S Derfuss T. Glatiramer acetate treatment normalizes deregulated microRNA expression in relapsing remitting multiple sclerosis. PLoS ONE. (2011) 6:e24604. 10.1371/journal.pone.0024604 21949733 PMC3174971

[88] Vereide DT Seto E Chiu YF Hayes M Tagawa T Grundhoff A. Epstein-Barr virus maintains lymphomas via its miRNAs. Oncogene. (2014) 33:1258–64. 10.1038/onc.2013.71 23503461 PMC3690170

[89] Seto E Moosmann A Gromminger S Walz N Grundhoff A Hammerschmidt W. Micro RNAs of Epstein-Barr virus promote cell cycle progression and prevent apoptosis of primary human B cells. PLoS Pathog. (2010) 6:e1001063. 10.1371/journal.ppat.1001063 20808852 PMC2924374

[90] Teymoori-Rad M Mozhgani SH Zarei-Ghobadi M Sahraian MA Nejati A Amiri MM. Integrational analysis of miRNAs data sets as a plausible missing linker between Epstein-Barr virus and vitamin D in relapsing remitting MS patients. Gene. (2019) 689:1–10. 10.1016/j.gene.2018.12.004 30552979

[91] Angelini DF Serafini B Piras E Severa M Coccia EM Rosicarelli B. Increased CD8+ T cell response to Epstein-Barr virus lytic antigens in the active phase of multiple sclerosis. PLoS Pathog. (2013) 9:e1003220. 10.1371/journal.ppat.1003220 23592979 PMC3623710

[92] Drosu NC Edelman ER Housman DE. Could antiretrovirals be treating EBV in MS? A case report. Mult Scler Relat Disord. (2018) 22:19–21. 10.1016/j.msard.2018.02.029 29510325 PMC6100748

[93] Hassani A Corboy JR Al-Salam S Khan G. Epstein-Barr virus is present in the brain of most cases of multiple sclerosis and may engage more than just B cells. PLoS ONE. (2018) 13:e0192109. 10.1371/journal.pone.0192109 29394264 PMC5796799

[94] Levin LI Munger KL Rubertone MV Peck CA Lennette ET Spiegelman D. Temporal relationship between elevation of epstein-barr virus antibody titers and initial onset of neurological symptoms in multiple sclerosis. JAMA. (2005) 293:2496–500. 10.1001/jama.293.20.2496 15914750

[95] Moreno MA Or-Geva N Aftab BT Khanna R Croze E Steinman L. Molecular signature of Epstein-Barr virus infection in MS brain lesions. Neurol Neuroimmunol Neuroinflamm. (2018) 5:e466. 10.1212/NXI.0000000000000466 29892607 PMC5994704

[96] Chen C Zhou Y Wang J Yan Y Peng L Qiu W. Dysregulated MicroRNA Involvement in Multiple Sclerosis by Induction of T Helper 17 Cell Differentiation. Front Immunol. (2018) 9:1256. 10.3389/fimmu.2018.01256 29915595 PMC5994557

[97] Dolati S Marofi F Babaloo Z Aghebati-Maleki L Roshangar L Ahmadi M. Dysregulated network of mirnas involved in the pathogenesis of multiple sclerosis. Biomed Pharmacother. (2018) 104:280–90. 10.1016/j.biopha.2018.05.050 29775896

[98] Gourzones C Gelin A Bombik I Klibi J Verillaud B Guigay J. Extra-cellular release and blood diffusion of BART viral micro-RNAs produced by EBV-infected nasopharyngeal carcinoma cells. Virol J. (2010) 7:271. 10.1186/1743-422X-7-271 20950422 PMC2974674

